# Seroepidemiology of trachoma in a low prevalence region receiving annual mass azithromycin distribution in Maradi, Niger

**DOI:** 10.1371/journal.pntd.0012727

**Published:** 2024-12-09

**Authors:** Abdou Amza, Boubacar Kadri, Beido Nassirou, Ahmed Arzika, Elisabeth Gebreegziabher, Huiyu Hu, Lina Zhong, Cindi Chen, Danny Yu, Thomas Abraham, YuHeng Liu, Karana Wickens, Thuy Doan, Diana Martin, Benjamin F. Arnold, Thomas M. Lietman, Catherine E. Oldenburg

**Affiliations:** 1 Programme National de Santé Oculaire, Niamey, Niger; 2 Faculté des Sciences de la Santé, Université Abdou Moumouni de Niamey, Niger; 3 Centre de Recherche et d’Intervention en Santé Publique, Niamey, Niger; 4 Francis I. Proctor Foundation, University of California, San Francisco, California, United States of America; 5 Department of Epidemiology & Biostatistics, University of California, San Francisco, California, United States of America; 6 Centers for Disease Control and Prevention, Atlanta, Georgia, United States of America; 7 Department of Ophthalmology, University of California, California, United States of America; University of Connecticut, UNITED STATES OF AMERICA

## Abstract

**Background:**

Trachoma programs use the indicator trachomatous inflammation-—follicular (TF) to monitor indication for and response to treatment for trachoma at the district level. Alternative indicators, including serologic markers, are increasingly being evaluated for trachoma surveillance. We evaluated seroprevalence of IgG antibodies to the Pgp3 antigen in two districts in Maradi, Niger thought to have low TF prevalence.

**Methods:**

Data were collected as part of the baseline assessment of the Azithromycin Reduction to Reach Elimination of Trachoma (ARRET) trial in September 2021. A random sample of 80 communities was selected from Mayahi and Guidan Roumdji districts, both of which had TF prevalence <20% at their most recent trachoma impact survey in 2018. A random sample of 50 children per community was sampled. We collected field grades, conjunctival swabs for processing PCR for ocular *Chlamydia trachomatis*, and dried blood spots for serologic assessment.

**Results:**

Of 3,994 children sampled in 80 communities, 49% were female and median age was 4 years. Overall TF prevalence was 4.6% (95% CI 3.5 to 5.8%) and trachomatous inflammation—intense (TI) prevalence was 0.6% (95% 0.3 to 0.9%). The prevalence of ocular chlamydia was 0.03% (95% CI 0.08%). Seroprevalence for Pgp3 antibodies was 6.3% (95% CI 5.5 to 7.1%) in 1–9-year-olds and 3.7% (95% CI 2.9 to 4.4%) in 1–5-year-olds. TF and Pgp3 seroprevalence were better correlated in 1–5-year-olds (correlation coefficient 0.29) compared to 1–9-year-olds (correlation coefficient 0.09).

**Conclusions:**

In this low trachoma prevalence setting in Niger, seroprevalence of antibodies to Pgp3 were consistent with little ongoing transmission of *C*. *trachomatis*.

## Author summary

Trachoma is the leading cause of infectious blindness globally and is caused by the bacteria *Chlamydia trachomatis*. Trachoma programs use the indicator trachomatous inflammation—follicular (TF) to monitor the progress of trachoma control efforts in endemic regions. However, TF is an imperfect indicator of trachoma transmission, and alternative indicators may be useful for monitoring trachoma. We evaluated the prevalence of TF, *C*. *trachomatis* as measured by PCR, and serologic markers of trachoma in an area of Niger with low prevalence of TF. We found little evidence of ongoing transmission of *C*. *trachomatis*, as evidenced by low prevalence of C. trachomatis and low seroprevalence of trachoma markers, suggesting that ongoing antibiotic distribution for trachoma may not be required in this region.

## Introduction

The clinical sign trachomatous inflammation—follicular (TF) in children aged 1–9 years is used to monitor trachoma prevalence in endemic evaluation units (roughly equivalent to a district). [1] In evaluation units with TF prevalence ≥ 10%, at least 3 rounds of annual mass drug administration with azithromycin are offered. In evaluation units with 5–9.9% TF prevalence, one round is offered. Communities with low prevalence of TF may have little ocular *Chlamydia trachomatis*, the causative organism of trachoma, and thus may not benefit from repeated rounds of azithromycin treatment.

In low prevalence settings, alternative indicators that allow for assessment of transmission of ocular chlamydia may be useful for guiding ongoing treatment decisions. IgG antibodies to *C*. *trachomatis* antigens have been proposed as an alternative indicator of past ocular *C*. *trachomatis* infection that can provide insight into transmission. [2,3] Antibodies to *C*. *trachomatis* are measured using dried blood spots, which are relatively easily collected in the field and transported without a cold chain, allowing them to be incorporated into existing surveillance surveys. Here, we evaluated seroprevalence of IgG antibodies to the Pgp3 antigen in two districts in Maradi, Niger which had approximately 15% prevalence of active trachoma at the most recent trachoma impact survey and met criteria for 3 to 5 additional rounds of mass azithromycin distribution. We report baseline measures of trachoma indicators for communities participating in the Azithromycin Reduction to Reach Elimination of Trachoma (ARRET), a cluster randomized trial evaluating whether mass azithromycin distribution can be discontinued in districts with TF prevalence <20% per the most recent trachoma impact survey. [4] Baseline trachoma indicators, including TF, ocular *C*. *trachomatis*, and Pgp3 seroprevalence, were collected to characterize ocular chlamydia transmission in the study communities. We anticipated that there would be little serologic evidence of recent *C*. *trachomatis* transmission in these communities given the low anticipated TF prevalence.

## Methods

### Ethics statement

The ARRET trial is registered at clinicaltrials.gov (NCT04185402) and complete methods have been previously reported for the trial. [4] This study was reviewed and approved by the Institutional Review Board at the University of California, San Francisco in the United States and the Comité National d’Ethique pour la Recherche en Santé in Niamey, Niger. Verbal consent was obtained from the guardian of each study participant prior to any study activities. CDC staff were determined to be non-engaged in human subjects research and did not interact with study participants or have access to identifying information.

This study was conducted in two evaluation units (Mayahi and Guidan Roumdji) in Maradi, Niger in September 2021. Maradi is located in southern Niger, a landlocked country in the Sahel region of sub-Saharan Africa. To be eligible for the study, districts had to have a TF prevalence below 20% per the most recent trachoma impact survey. In 2018, TF prevalence was 16.8% in Mayahi and 15.1% in Guidan Roumdji based on a trachoma impact survey conducted per World Health Organization (WHO) guidelines. These evaluation units received 8 rounds of mass azithromycin distribution from 2002 through 2020. The most recent azithromycin distribution prior to sample collection was in March 2020, 18 months before sample collection. The districts had received two mass azithromycin distributions since the most recent trachoma impact survey in 2018. In September 2021, 80 communities in the two study districts were randomly selected to participate in the ARRET trial, a cluster randomized trial evaluating continued azithromycin distribution versus stopping azithromycin distribution in districts with TF < 20% per the most recent trachoma impact survey. [5]

Prior to the baseline assessment, an enumerative census was conducted in all study communities. During the baseline assessment for the trial, a simple random sample of 50 children aged 0–9 years per community was selected from the census for examination. The sample size was based on the overall sample size calculation for the trial. [4] A trachoma survey with Tropical Data-trained graders was conducted among all selected children. [6] Field grades were assigned per WHO simplified trachoma grading, including TF and trachomatous inflammation—intense (TI). A conjunctival swab was collected from the right everted eyelid of each child who was examined. Conjunctival swabs were pooled in pools of 5 as previously described and were processed using polymerase chain reaction (PCR) for *C*. *trachomatis* using loop-mediated isothermal amplification (Atila Biosystems, Sunnyvale, CA). [7] This is a commercial assay that has been validated for conjunctiva, vaginal, and cervical swabs collected as dry swabs. The limit of detection for this assay is 10 copies of Ct per sample. Finger prick blood was collected onto TropBio filter paper (CellLabs, Sydney, Australia) and tested for IgG antibodies to Pgp3 in children aged 1–9 years using a multiplex bead assay on the Luminex platform as previously described. [8] Antibodies were not assessed in children < 1 year due to the presence of maternal antibodies. The cutoff for seropositivity was determined to be median fluorescence intensity minus background (MFI-bg) > 163 using a receiver operating characteristic curve cutoff from reference samples. [9] PCR testing was performed at the University of California, San Francisco and serologic testing was performed at the Centers for Disease Control and Prevention in Atlanta, GA.

We evaluated the distribution of TF, TI, ocular chlamydia, and seroprevalence of Pgp3 at the community level in children aged 1–9 years, 18 months after the most recent azithromycin distribution. The mean prevalence of each trachoma indicator was calculated at the community level and 95% confidence intervals (CI) were estimated. We estimated correlation coefficients for community-level correlations between TF and Pgp3. We originally planned to evaluate correlation coefficients for TF and Pgp3 with ocular *C*. *trachomatis* prevalence, but such analyses were not possible due to the extremely low prevalence of ocular *C*. *trachomatis*. Age-seroprevalence curves for Pgp3 were estimated by estimating the pooled prevalence of each antigen in 1-year age bands. Because seroprevalence in older children reflects historical transmission, we also evaluated seroprevalence in children aged 1–5 years. The seroconversion rate per 100 child-years in 1–5-year-olds, assuming no seroreversions, was estimated using a generalized linear model with a complementary log-log link using age as the offset with standard errors accounting for clustering at the community level. [10–12] Seroconversion rates were estimated in the younger age range to reduce influence of historical transmission in older children. All analyses were run in Stata version 17.0 (StataCorp, College Station, TX).

## Results

In 80 communities randomly selected to participate in the study, guardians of 4,006 children consented to their child’s participation in sample collection with no refusals. Of these, 3,994 children were in the 0–9-year age range and had field grades and swabs collected, and 3,602 were in the 1–9-year age range and had dried blood spots collected for serology. Clinical trachoma and *C*. *trachomatis* prevalence estimates were available for all 80 communities. Dried blood spots were lost for 49 children due to mold, and thus 3,553 samples in 80 communities were tested and included in serologic analyses. Of the consented children, 49% were female, and median age was 54 months (IQR 28 to 77 months; **Table 1**).

**Table 1:** Demographic characteristics of included children.

	Overall Sample	Serology Sample
N	3,994	3,553
Female sex, N (%)	1,954 (48.9%)	1,758 (49.5%)
Age, years, N (%)		
0	366 (9.2%)	0
1	464 (11.6%)	410 (11.5%)
2	468 (11.7%)	468 (13.2%)
3	527 (13.2%)	525 (14.8%)
4	441 (11.0%)	438 (12.3%)
5	504 (12.6%)	500 (14.1%)
6	440 (11.0%)	433 (12.2%)
7	317 (7.9%)	316 (8.9%)
8	304 (7.6%)	302 (8.5%)
9	163 (4.1%)	161 (4.5%)

**Fig 1:**
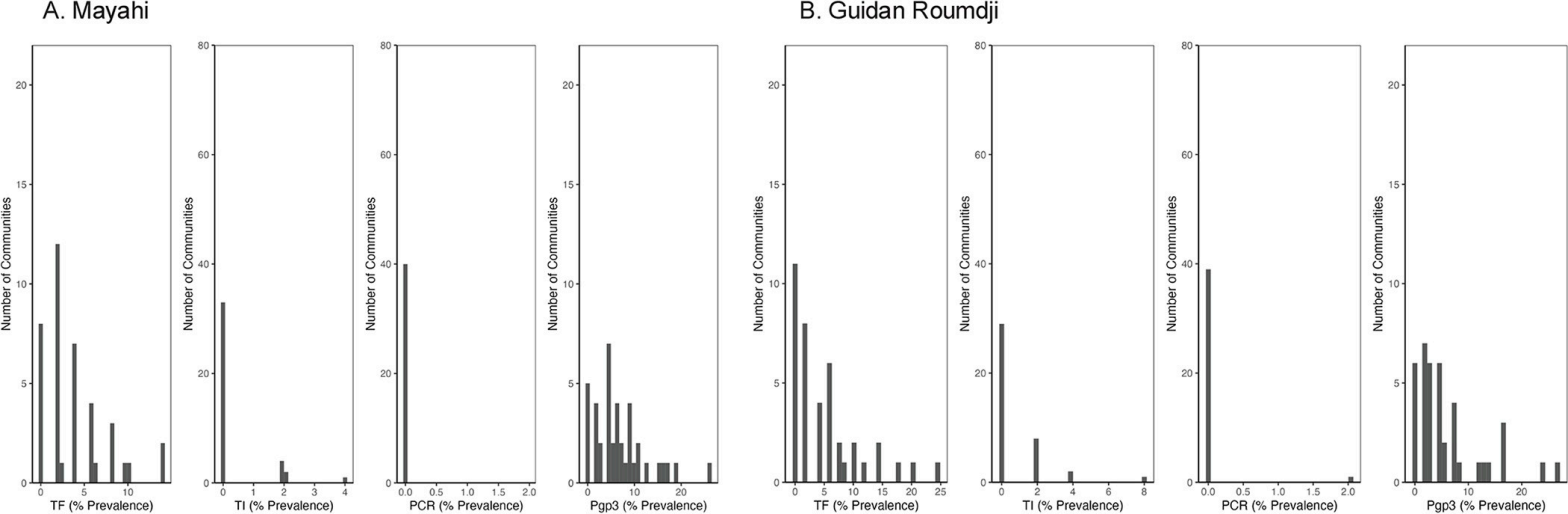
Histograms showing community-level distribution in 1-9-year-olds of trachomatous inflammation–follicular (TF), trachomatous inflammation–intense (TI), ocular Chlamydia trachomatous infection by PCR, and seroprevalence to Pgp3 in Mayahi (Panel A) and Guidan Roumdji (Panel B).

Overall mean cluster TF prevalence was 4.6% (95% CI 3.5 to 5.8%) and ranged from 0 to 24% in individual communities (**Fig 1**). TF prevalence was 3.9% (95% CI 2.7 to 6.2%) in Mayahi and 5.4% (3.4 to 7.3%) in Guidan Roumdji. Mean cluster TI prevalence was 0.6% (95% CI 0.3 to 0.9%): 0.4% (95% CI 0.1 to 0.7%) in Mayahi and 0.8% (95% CI 0.3 to 1.3%) in Guidan Roumdji. Only a single pool was positive by PCR for *C*. *trachomatis*, leading to an estimated ocular chlamydia prevalence of 0.03% (95% CI 0 to 0.08%). Seroprevalence with Pgp3 was 6.3% (95% CI 5.5 to 7.1%) in 1–9-year-olds and 3.7% (95% CI 2.9 to 4.4%) in 1–5-year-olds. Table 2 shows seroprevalence and seroconversion rates by district and age group. There was no evidence of correlation between TF prevalence and Pgp3 in 1–9-year-olds (correlation coefficient 0.09, 95% CI -0.22 to 0.39) and weak correlation between Pgp3 and TF prevalence in 1–5-year-olds (correlation coefficient 0.29, 95% CI -0.04 to 0.62).

**Table 2:** Seroprevalence for Pgp3 by district in Maradi, Niger.

	Mayahi Mean (95% CI)	Guidan Roumdji Mean (95% CI)
Age (years)		
1	0.4% (0 to 1.3%)	0.7% (0 to 2.0%)
2	1.8% (0 to 3.6%)	2.7% (0.6 to 4.8%)
3	3.8% (1.6 to 6%)	4.7% (1.8 to 7.5%)
4	5.3% (2.2 to 8.4%)	3.4% (0.9 to 5.9%)
5	8.1% (4.7 to 11.6%)	4.3% (1.7 to 6.9%)
6	8.2% (4.5 to 12%)	5.9% (2.7 to 9.2%)
7	15.2% (10 to 20.4%)	13.2% (6.9 to 19.4%)
8	11.3% (6.2 to 16.4%)	10.6% (5.5 to 15.7%)
9	24.1% (14.6 to 33.5%)	12.3% (4.8 to 19.9%)
Seroconversion rate		
1-9-year-olds	1.7 per 100 child-years (1.3 to 2.2)	1.3 per 100 child-years (0.9 to 1.8)
1-5-year-olds	1.3 per 100 child-years (0.9 to 2.0)	1.1 per 100 child-years (0.7 to 1.6)

**Fig 2:**
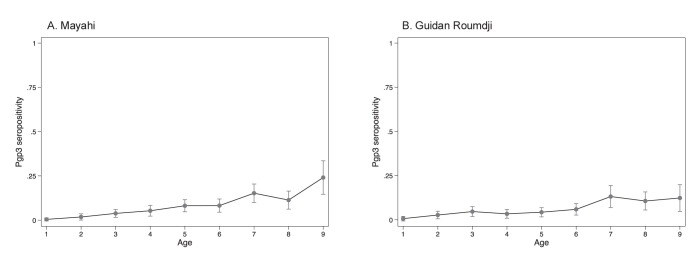
Age-seroprevalence curve for seropositivity to Pgp3 by age in years among children aged 1–9 years in Mayahi (Panel A) and Guidan Roumdji (Panel B).

Seroprevalence for Pgp3 was 0.5% in 1-year-olds (95% CI 0 to 1.3%; **Fig 2**), 6.3% in 5-year-olds (95% CI 4.1 to 8.4%), and 18.4% in 9-year-olds (95% CI 12.2 to 24.6%; **Fig 2**). The estimated seroconversion rate (force of infection) based on Pgp3 was 1.2 per 100 child-years (95% CI 0.9 to 1.6) in 1–5-year-olds.

## Discussion

In this study using data from a population representative sample of two evaluation units in Maradi, Niger that had trachoma prevalence in 2018 suggesting they required 3–5 years of mass azithromycin distribution, we found little evidence of ongoing trachoma transmission. Despite missing one round of mass azithromycin distribution in 2021 due to COVID-19 restrictions, mean TF prevalence was below the threshold for control, suggesting that trachoma transmission is very low in this setting despite receiving only two rounds of mass azithromycin distribution since the last trachoma impact survey. We found very little evidence of ocular chlamydia infection, with only a single positive pool by PCR. This finding is consistent with the hypothesis that very low TF prevalence areas have no ongoing *C*. *trachomatis* transmission.

Antibodies to the *C*. *trachomatis* antigen Pgp3 were consistent with TF and PCR results, offering limited evidence of ongoing transmission of ocular chlamydia. A previous pooled analysis evaluating the relationship between Pgp3 seroepidemiology, PCR positivity, and clinical trachoma in 14 cohorts demonstrated strong correlation between seroprevalence and seroconversion and PCR prevalence. [13] That analysis found that a seroprevalence threshold of 13.5% or seroconversion rate of 2.75 per 100 person-years had high sensitivity for identification of clusters with ocular *C*. *trachomatis* infection. In the present analysis, both seroprevalence and the seroconversion rate are consistent with an absence of ocular chlamydia, consistent with the previous analysis.

The correlation between TF and Pgp3 seroprevalence was stronger in the 1–5-year-old age group compared to the 1–9-year-old age group, although TF and Pgp3 were only weakly correlated in 1–5-year-olds. Because antibody durations are durable and seroreversion is rare [14], monitoring serologic markers in 1–5-year-olds may be more reflective of recent transmission patterns compared to including older children. In the present analysis, the higher seroprevalence in children over age 6 likely reflects historical transmission of ocular chlamydia. Trachoma transmission was likely considerably higher when these children were younger and exposed to ocular chlamydia, as evidenced by the higher TF prevalence documented in the 2018 trachoma impact survey.

A “wait and watch” approach has been proposed for districts with TF prevalence just above the 5% threshold at trachoma surveillance surveys. [15] In Amhara, Ethiopia, two evaluation units that were just above the 5% threshold during a trachoma surveillance survey were enrolled in a wait and watch program in which they did not receive mass azithromycin distribution but were re-surveyed one year later to assess indicators of trachoma, including TF and infection prevalence as well as Pgp3 serology. [15] The Ethiopian study demonstrated little evidence of trachoma under the wait and watch strategy and no evidence of recrudescence. Given that the districts in the present study had not received mass azithromycin distribution for 18 months, the present results support the wait and watch strategy. These data are from the baseline assessment from a cluster randomized trial that randomizes communities to stopping compared to continuing annual mass drug administration when they are in this grey zone and are expected to provide definitive evidence supporting this strategy. [5]

This study included only two districts in Niger and thus generalizability to other settings may be limited. However, as previously discussed, the correlation between TF prevalence and Pgp3 seroprevalence and seroconversion was highly consistent with a large previous analysis. [13] The prevalence of TF was lower than anticipated during the planning of the trial and was right at the threshold for elimination as a public health problem. While these results suggest that there is no ongoing ocular *C*. *trachomatis* transmission, they may not be generalizable to other settings with higher TF prevalence. These results suggest that the inclusion of alternative indicators for trachoma, including infection and serology, may help increase confidence that there is truly no ongoing transmission. However, additional costs and logistics associated with enhanced trachoma surveillance may limit the settings in which such a strategy is feasible.

These results demonstrated little evidence of ongoing trachoma transmission in two previously endemic districts in Maradi, Niger. At the time of survey, the districts had not received azithromycin for 18 months, but TF prevalence was far below that of the most recent trachoma surveillance survey. Given lack of evidence supporting ongoing trachoma transmission, these results suggest that continued azithromycin distribution may not be required in districts in which trachoma impact surveys indicate 3 to 5 additional rounds of treatment.

## Supporting information

S1 Checklist CONSORT checklist. (DOCX)
